# Expectations of occupational health physicians and nurses regarding the management of evidence-based practice in occupational health care: a qualitative study

**DOI:** 10.1108/IJHCQA-05-2026-0150

**Published:** 2026-09-09

**Authors:** Kati Päätalo, Kirsi Koivunen

**Affiliations:** Oulu University of Applied Sciences, Oulu, Finland

**Keywords:** Process management, Evidence-based practice, Private healthcare, Management, Quality management, Nursing quality

## Abstract

**Purpose:**

The aim of this qualitative study is to describe the expectations of occupational health physicians and nurses regarding managerial support in evidence-based practice in occupational health care.

**Design/methodology/approach:**

Qualitative data were collected from Finnish physicians and nurses working in occupational health services in Finland (*n* = 126). The data were analysed using inductive content analysis.

**Findings:**

As a result, three main categories were revealed: (1) knowledge management in occupational health care, (2) management of operative processes in occupational health care and (3) supportive human resource management in occupational health care.

**Originality/value:**

In conclusion, supporting evidence-based practice in occupational health care requires investment in the development of staff competence. Evidence-based occupational health should be integrated into a culture at an attitudinal and practical level. Clear evidence-based guidelines, uniform processes and compliance with these play a key role. Promoting evidence-based occupational health involves knowledge management, operative process management and supportive human resource management skills.

## Introduction

To ensure the efficacy of the service system, occupational health services (OHS) are expected to be based on the best available research evidence (Cabling *et al.*, 2025). Evidence-based practice (EBP) involves “…giving consideration to the best available evidence; the context in which the care is delivered; client preference; and the professional judgement of the health professional” (Pearson *et al.*, 2005). The JBI Model of Evidence-Based Healthcare (JBI Model of EBHC) provides a framework for the generation, synthesis, transfer, and implementation of evidence in healthcare (Jordan *et al.*, 2019, Lockwood, 2017). The JBI Model of EBHC (Jordan *et al.*, 2019; Lockwood, 2017) consists of five segments: global health (knowledge need), evidence generation (well-designed studies), evidence synthesis (guidelines, evidence summaries, systematic reviews), evidence transfer (dissemination of knowledge to health professionals and systems), and evidence implementation (activities which engage with evidence). In the model, EBP is seen as part of evidence implementation (Jordan *et al.*, 2019).

In occupational health service (OHS) practices, evidence forms the basis for decision-making that involves individual service users, workers, and their health and safety. Evidence-based decision-making also occurs at the workplace level, where enterprises act as clients. The use of evidence in OHS decision-making will promote efficiency and, for example, reduce the number of occupational incidents and diseases (Stockwell *et al.*, 2022). Successful evidence-based practice in occupational health care depends on organisational support, and the competence and attitude of practitioners (Ruotsalainen *et al.*, 2024). It also requires the development of more effective methods and tools for sharing evidence on occupational safety and health within organisations (Stockwell *et al.*, 2022).

Melnyk *et al.* (2021) and Furuki *et al.* (2023) found that organisations that promote a culture of openness and embrace EBP implementation can improve their practitioners' EBP competence, beliefs, and adoption, leading to consistent EBP use and, ultimately, improved care outcomes. Promoting a positive EBP culture is also important in occupational health organisations (Stockwell *et al.*, 2022; Cabling *et al.*, 2025). Management plays a major role in evidence transfer and implementation at an organisational level, supporting practitioners' evidence-based practice and decision-making (Bianchi *et al.*, 2018; Castiglione, 2020). The core competences of health care managers are the ability to analyse issues critically, use information creatively, anticipate developments and their consequences, and respond to future challenges (Kantanen *et al.*, 2015).

EBP is a key area of knowledge management for managers (Lunden *et al.*, 2021). Knowledge-based human resource management includes recruiting and selecting staff based on their knowledge and skills, assessing performance, competency-based rewards, motivating staff, supporting career planning, and providing training and courses (Gunawan *et al.*, 2019; Liu *et al.*, 2022). Knowledge management includes organising the acquisition and sharing of knowledge, generating new knowledge, and putting it into practice effectively (Popa *et al.*, 2018; Sibbald *et al.*, 2016). Today's managers are expected to have more skills to support, motivate, coach and enable the professional development of staff (Nurmeksela *et al.*, 2020). The combination of KM (knowledge management) and EBP entails certain principles for enhancing competencies: learning to apply new knowledge and innovation within organisations in a collaborative manner (Sibbald *et al.*, 2016).

Although research on EBP has been available for several decades, the practice has not been implemented successfully by nursing management, and the role of managers is as invisible in KM as it is in EBP (Lunden *et al.*, 2021). Nurses need support and resources at an organisational level to implement EBP (Renolen *et al.*, 2018; Bianchi *et al.*, 2018). Managers act as role models for sharing knowledge and promoting a culture of knowledge sharing in organisations (Ayatollahi and Zeraatkar, 2020) and play a key role in implementing evidence-based practice in terms of providing a supportive culture and environment. Managers can also influence their staff's EBP competence (knowledge, skills, and attitude) and the implementation of EBP (Guerrero *et al.*, 2020) and lead their staff's implementation of EBP through close interaction (Bianchi *et al.*, 2018), participating significantly in the supervision and guidance of care (Gifford *et al.*, 2018) and acquiring the necessary resources and training opportunities (Williams *et al.*, 2020).

Managers need to have basic knowledge of EBP and be aware of and address the barriers to its implementation. In some reviews (Kakemam *et al.*, 2020; González-García *et al.*, 2021) that summarise health care leadership and management competencies, evidence-based practice is recognised as one of the core competences. This competence is described in terms of evidence-informed decision-making, including evidence appraisal, evidence application and decision making, evaluation of decisions (Kakemam *et al.*, 2020) and research and evidence-based practice (González-García *et al.*, 2021). In the Management Competency Assessment Tool (MCAP Tool) for health service managers, evidence is one of the competence areas that is measured (Howard *et al.*, 2018).

In a scoping review of Koivunen *et al.* (2024), the competences of frontline nursing leaders in evidence-based healthcare were identified and classified according to the JBI Model of EBHC. Evidence implementation ended up being the strongest segment. This included the ability to evaluate the outcomes of EBP changes, the ability to evaluate one's own practice, the ability to implement recommendations in practice, the ability to support the implementation of EBP changes, the ability to use evidence in practice, and the ability to use evidence in leadership. The evidence transfer segment emerged as the weakest, including the ability to encourage staff to read evidence and share knowledge.

Only a few studies have focused on evidence-based practice in the context of occupational health. Verbeek (2018), Teufer *et al.* (2019) and Cabling *et al.* (2025) suggest that EBP is underutilised in OHS, and that recommendations for improving occupational health and safety are often based on the opinions of individuals rather than direct consultation of more rigorous sources of evidence. Earlier studies provide information on the attitudes of nurses and doctors towards evidence-based guidelines (Kinnunen-Amoroso, 2013), physicians' attitudes towards evidence-based occupational health and clinical practice guidelines (Heselmans *et al.*, 2010), and the development of EBP in relation to attitudes, knowledge, and use (Brämberg *et al.*, 2017). The general attitude of nurses and doctors towards evidence-based occupational health and guidelines is positive (Kinnunen-Amoroso, 2013; Heselmans *et al.*, 2010; Cabling *et al.*, 2025). Doctors were more familiar with EBM (evidence-based medicine) and EBG (evidence-based guidelines) than nurses. Both professional groups considered that there are too few guidelines aimed at occupational health (Kinnunen-Amoroso, 2013). Barriers to applying existing evidence to practice included time constraints, a lack of EBM skills (Heselmans *et al.*, 2010) and a lack of training in searching for guidelines or scientific information (Kinnunen-Amoroso, 2013). EBGs were considered useful for decision-making in daily practice, although they were considered impractical and difficult to use (Kinnunen-Amoroso, 2013). EBGs need to be short, simple, and easy to read (Kinnunen-Amoroso, 2013). Legal, regulatory, business and cultural factors influence OHS-related decision-making and are often prioritised over OHS evidence (Stockwell *et al.*, 2022; Cabling *et al.*, 2025).

As regards managing evidence-based practice (Brämberg *et al.*, 2017), managers welcomed EBP and saw their role as supporting practitioners by giving them opportunities for training, conference participation or clinical guideline development. Similarly, OHS managers requested brief, easy-to-use and cost-effective guidelines to facilitate EBP. However, practitioners noted that OHS management could adopt a more open learning culture and support competence development in order to promote a culture of health and safety (Cabling *et al.*, 2025). Brämberg *et al.* (2017) and Stockwell *et al.* (2022) have concluded that managers need to be educated to support EBP at the organisational level by creating an infrastructure for EBP in OHS.

These previous studies provide valuable insights into attitudes, barriers and factors that favour evidence-based occupational health care. Organisational and management support plays a major role in evidence-based practice, so it is necessary to focus on managerial support and the role of managers in the context of multiprofessional occupational health care.

## Aim

The aim of this study is to describe the expectations of occupational health physicians and nurses regarding managerial support for evidence-based practice in occupational health care.

## Methods

Qualitative data was collected as part of a national electronic survey conducted in Finland between November 2020 and August 2021. The survey was conducted as part of a research project entitled “Evidence-based occupational health care in Finland” (Päätalo and Ruotsalainen, 2022), funded by the Finnish Work Environment Fund. The questionnaire was completed by a sample of 524 Finnish physicians and nurses working in OHS. A total of 126 respondents, 87 nurses and 39 doctors, responded to the open question: “What kind of support do you expect from your manager in relation to evidence-based practice?”

The data were analysed using inductive content analysis (Graneheim and Lundman, 2004; Elo and Kyngäs, 2008; Schreier, 2012). The fundamental statements that answered the research question were collected into lists and coded (170 codes). From this code list, statements presenting similar content were classified into eight subcategories and three main categories. The categories were named based on their content.

Following the principle of informed consent, survey respondents were informed about the purpose of the study, anonymity, voluntariness, confidentiality of data, and the contact details of the researchers and organisations cooperating in the study (Oulu University of Applied Sciences, Nursing Research Foundation, Finnish Occupational Physicians’ Association, Finnish Association of Occupational Health Nurses). A data privacy notice compliant with EU GDPR requirements was publicly available.

Trustworthiness was assessed using the evaluation criteria for qualitative studies (credibility, dependability, transferability, and confirmability) and content analysis (Lincoln and Guba, 1985; Polit and Beck, 2008; Elo *et al.*, 2014). To ensure credibility, a principle of data saturation was applied (Polit and Beck, 2008). Following this principle, a sense of closure was attained: during the analysis process, it was observable that the same themes were repeated in the data. Based on this observation, the amount of data was evaluated as sufficient. According to Elo *et al.* (2014), the trustworthiness of content analysis results depends on the availability of rich, appropriate, and well-saturated data. In terms of dependability, the data were collected anonymously via an electronic survey without direct contact with the researcher. The data analysis was carried out without presumptions. From the point of view of transferability, the data were collected at a national level in a single country. The results can be transferred to other settings or groups, taking cultural differences into account. To ensure confirmability, the characteristics of the data are described and quotations are presented to authenticate the results of this study. An inductive content analysis process is also presented in detail.

## Results

The expectations of physicians and nurses specialising in occupational health regarding managerial support for evidence-based practice were divided into three main categories: (1) Knowledge management in occupational health care, (2) Management of operative processes in occupational health care, and (3) Supportive human resource management in occupational health care (Table 1).

**Table 1:** Expectations of occupational health physicians and nurses regarding managerial support for evidence-based practice

Subcategory	Main category
Enabling training and education	*Knowledge management in occupational health care*
Active inclusion in research and development	
Maintaining culture of sharing competence and knowledge	
Supervising work in consistent, evidence-based practices	*Managing operative processes in occupational health care*
Knowledge and competence of occupational health care services	
Enabling personal professional development	*Supportive human resource management in occupational health care*
Ensuring resources	
Positive attitude and leading by example	

## Knowledge management in occupational health care

Knowledge management in occupational health care consists of: (1) enabling training and education, (2) active inclusion in research and development, and (3) maintaining a culture of sharing competence and knowledge.

As an important part of knowledge management, managers are expected to *enable training and education* to support evidence-based practice. A positive attitude towards education, encouragement, permission, and opportunity for training is expected. Training should be regular, and employees should be supported in identifying training needs. Managers are also expected to identify these needs and provide training. Despite pressure for monetary gain in occupational health services, training should be prioritised.

An organisation should offer *active inclusion in research and development*. This takes the form of support for theses and research-based development activities. Employees should have the space and resources to participate in research and development in the field of occupational health care research. Research meetings, such as journal clubs, are recommended.

Knowledge management also includes *maintaining a culture of sharing competence and knowledge*. Issues concerning research evidence in occupational health care methods should be discussed regularly, for example in joint meetings. Information sharing and discussion between professionals is important. Managers are expected to actively present evidence-based information in meetings and ensure knowledge sharing. Gathering and sharing up-to-date information is essential; managers should be aware of the latest information and actively share it.

An active approach and addressing the issue and, for example, reporting on new evidence-based issues. (Doctor 1)

In joint meetings, managers can share new studies that have been compiled for the organisation to communicate. I don't necessarily see it as the supervisor's job to seek out research information, but rather to pass it on. The development team, etc., could keep the information up to date. (Nurse 61)

Staying informed and bringing new ideas to the work community. (Nurse 32)

## Managing operative processes in occupational health care

Managing operative processes in occupational health care consists of: (1) supervising work to ensure that it complies with consistent, evidence-based practices, and (2) knowledge and competence of occupational health care services.

Occupational health practices should be evidence-based and consistent. Professionals should be guided to act on the basis of evidence and in a consistent manner to ensure quality of services. It is important to guide and support staff in changing their practices and basing them on evidence. Guidelines should be clear, simple and easy to use. Managers are expected to provide orientation, support and advice. They should also oversee and assess whether practices are consistent with evidence-based information.

In managing operative processes to ensure that they are evidence-based, the *knowledge and competence of occupational health care services* are of great importance. It was seen as important for managers to have practical competence, knowledge and skills regarding occupational health care. Practical competence and experience are needed to help staff solve problems concerning practical issues. For example, it was mentioned that a master's degree was applicable when combining research and practice.

Implementation of operations in everyday life and monitoring so that everyone adopts and operates in a consistent manner. (Nurse 15)

The manager should be the one to identify the latest issues and current changes and implement them in the everyday work of occupational health nurses. (Nurse 45)

That there is permission to do evidence-based work. Many occupational health care units sell (sic) customers some kind of activity to finance operations without evidence. This obscures the role of activities that have been proven to be effective. (Doctor 30)

Support as regards changing practices to make them more evidence-based. (Doctor 10)

## Supportive human resource management in occupational health care

Supportive human resource management consists of: (1) enabling personal professional development, (2) ensuring resources, and (3) maintaining a positive attitude and leading by example.

To support evidence-based practice, managers are expected to *enable personal professional development*. The supervisor should provide personal guidance, and sufficient time should be reserved for personal discussions. In performance appraisals, it is important to set personal goals and create a personal training plan. Supervisors should also map employees' competence in relation to evidence-based practice. It is important that the strengths of employees are supported. Giving feedback and rewarding achievements is also essential.

Supportive human resource management includes *securing resources*. In particular, the organisation should provide time and other necessary resources for competence development. This means, for example, using working time to read research publications and adopt new organisational guidelines. Employees should be encouraged and enabled to devote working time to study, using the necessary tools. It is important to allocate resources to development work and to ensure that employees have enough time and support to do high-quality work.

Managers are expected to have *a positive attitude and lead by example.* It is important that they understand the importance of development and evidence-based practice. The manager should act as an example and motivate employees to adopt evidence-based practices. Another important role they have is to bring new ideas to the work community and encourage renewal. Their role as positive enablers when it comes to experimenting with new ways of working is essential.

The manager has led the way in this matter in an exemplary manner. Matters are discussed and common practices are agreed upon. (Doctor 6)

The manager should better understand their own importance as an example and as a guide. (Nurse 84)

## Discussion

This study provided new insights into evidence-based practice and its management in the context of occupational health care. It focused on occupational health physicians and nurses. In physicians' work, the theoretical background is traditionally based on evidence-based medicine (Sackett *et al.*, 1996). Possibly due to this strong tradition, occupational health doctors seem to be more familiar with evidence-based medicine and evidence-based guidelines than nurses (Kinnunen-Amoroso, 2013). Recent models created for evidence-based healthcare, such as the JBI Model of EBHC (Jordan *et al.*, 2019; Lockwood, 2017), enhances the evidence-based framework common to all health care professionals. However, the historical and paradigmatical backgrounds need to be acknowledged in the management of evidence-based practice in multiprofessional occupational health care.

This study highlights the management competences necessary for successful management that supports evidence-based practice in the context of occupational health care. Three aspects are emphasised: (1) knowledge management, (2) managing operative processes, and (3) supportive human resource management. The expected managerial competences will be discussed within the framework of the JBI Model of EBHC. The model consists of global health, evidence generation, evidence synthesis, evidence transfer, and evidence implementation. The competences focus especially on the phases of evidence transfer and implementation, in line with the conception of the EBHC model (Jordan *et al.*, 2019). In the evidence transfer and implementation phase, the relevant actors are representatives of education and organisations (Jordan *et al.*, 2019). Within organisations, managers play a major role in evidence transfer and implementation (Bianchi *et al.*, 2018; Castiglione, 2020). During these stages, knowledge is disseminated among professionals and activities which engage with evidence are carried out (Jordan *et al.*, 2019).

Evidence transfer and implementation in occupational health care requires knowledge management, which divides the individual and community levels. Regarding the individual level, managers should provide employees with opportunities for training and involvement in research and development activities. At the work community level, managers are expected to create and maintain a culture of sharing competence and knowledge. It has been shown that managers' knowledge management competencies are critical for EBP implementation, as they mediate the effects of leadership and organisational support (Abou Hashish *et al.*, 2025). The starting point for knowledge management would appear to be straightforward, as the attitude of occupational health care employees towards evidence-based practice are noted to be positive (Heselmans *et al.*, 2010; Kinnunen-Amoroso, 2013; Brämberg *et al.*, 2017; Stockwell *et al.*, 2022; Cabling *et al.*, 2025).

In terms of managing operative processes, managers' practical competence and knowledge regarding occupational health care services was appreciated. In addition to management competence, professional competence is also needed to manage evidence-based occupational health care. Employees expect that their work will be supervised in terms of applying evidence-based consistent practices. In previous studies, there have been calls for brief, easy-to-use and cost-effective guidelines to facilitate EBP in daily work (Cabling *et al.*, 2025; Brämberg *et al.*, 2017; Kinnunen-Amoroso, 2013), which is a useful way of supervising employees to follow consistent practices. The manager's role is also to oversee and evaluate whether practice is in accordance with these guidelines and consistent practices. Brämberg *et al.* (2017) and Stockwell *et al.* (2022) conclude that evidence-based practice in occupational health care requires strong organisational and managerial support. It is notable that in occupational health care organisations, gaps in this support have been revealed (Brämberg *et al.*, 2017; Stockwell *et al.*, 2022; Cabling *et al.*, 2025).

In addition to managing competence and operative processes, there is a need for supportive human resource management. Personal professional development and resources should be ensured through management. The importance of sufficient time resources was emphasised, consistent with previous studies (Heselmans *et al.*, 2010). Furthermore, supportive human resource management includes renewal, encouragement and exemplary behaviour on the part of managers. Similarly, the review by Bianchi *et al.* (2018) reveals that nursing managers play an influential role in providing a supportive culture and environment for evidence-based practice. In the context of occupational health care, there is also a need for skill development to ensure the application of evidence in practice (Heselmans *et al.*, 2010).

The stages of evidence generation (well-designed studies) and evidence synthesis (guidelines, evidence summaries, systematic reviews) form the basis of evidence-based occupational health care. These stages are primarily the tasks of researchers and experts in the field (Jordan *et al.*, 2019). In terms of generating evidence-based research and its transfer to the practical sphere, it is notable that previous studies highlight the need to strengthen the transfer of evidence-based research to the practical sphere of occupational health care (EU-OSHA, 2014). Suggestions are also made for occupational health care researchers, who are encouraged to disseminate their research results more actively outside the scientific community (Jensen *et al.*, 2020). In the study by Kinnunen-Amoroso (2013), both nurses and doctors considered that there were too few evidence-based guidelines aimed at occupational health (Kinnunen-Amoroso, 2013). This study highlights the competences that managers need at the service producer level to ensure evidence-based practice. The latter is made possible by the evidence that is generated and synthesised, and therefore it seems that there is also a challenge as regards generating evidence that is focused on occupational health care, synthesising it and transferring it into practice so that it can be successfully implemented through management.

## Conclusions

In order to support evidence-based practice in occupational health care, managers must invest in the development of staff competence. Evidence-based activities require personal professional development, sufficient training, and opportunities to participate in research and development activities. At the community level, it is essential to have a culture that shares expertise. Managers should lead by example in evidence-based occupational health care, both in terms of attitude and practice, which also requires competence in the content of occupational health care services. At the organisational level, it is important to support the work with clear guidelines and uniform processes, and also to ensure that the work is carried out in accordance with them in practice. Occupational health care organisations should ensure that their managers have the necessary expertise in the areas of knowledge management, operative process management and supportive human resource management, so that they can promote evidence-based practices among their staff.
